# Amniotic membrane, a novel bioscaffold in cardiac diseases: from mechanism to applications

**DOI:** 10.3389/fbioe.2024.1521462

**Published:** 2024-12-20

**Authors:** Hossein Rayat Pisheh, Ahmad Darvishi, Seyed Saeid Masoomkhah

**Affiliations:** ^1^ Department of Tissue Engineering and Applied Cell Sciences, School of Advanced Medical Sciences and Technologies, Shiraz University of Medical Sciences, Shiraz, Iran; ^2^ Student Research Committee, Shiraz University of Medical Sciences, Shiraz, Iran; ^3^ School of Pharmacy, Shiraz University of Medical Sciences, Shiraz, Iran; ^4^ Department of Biomedical Engineering, Meybod University, Meybod, Iran

**Keywords:** amniotic membrane, cardiac diseases, biological scaffold, stem cells, regenerative medicine, cardiomyocytes

## Abstract

Cardiovascular diseases represent one of the leading causes of death worldwide. Despite significant advances in the diagnosis and treatment of these diseases, numerous challenges remain in managing them. One of these challenges is the need for replacements for damaged cardiac tissues that can restore the normal function of the heart. Amniotic membrane, as a biological scaffold with unique properties, has attracted the attention of many researchers in recent years. This membrane, extracted from the human placenta, contains growth factors, cytokines, and other biomolecules that play a crucial role in tissue repair. Its anti-inflammatory, antibacterial, and wound-healing properties have made amniotic membrane a promising option for the treatment of heart diseases. This review article examines the applications of amniotic membrane in cardiovascular diseases. By focusing on the mechanisms of action of this biological scaffold and the results of clinical studies, an attempt will be made to evaluate the potential of using amniotic membrane in the treatment of heart diseases. Additionally, the existing challenges and future prospects in this field will be discussed.

## Graphical Abstract

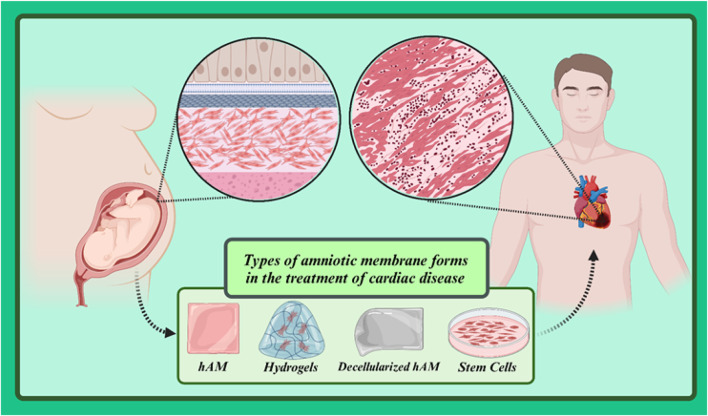

## 1 Introduction

Cardiovascular diseases (CVDs) are one of the main causes of death worldwide, and despite significant advances in diagnosis and treatment, there is a need for new and effective treatment approaches (Sansonetti et al., 2024). In recent years, researchers have turned their attention to the use of natural biomaterials. Statistics show a bleak picture: in 2019 alone, around 18.6 million people worldwide succumbed to cardiovascular diseases (Gaziano, 2022). With an aging population, the prevalence of cardiovascular disease is expected to continue to increase. In addition, unhealthy lifestyles contribute to risk factors such as obesity, high cholesterol and high blood pressure, making younger people more susceptible to heart disease (writing committee of the report on cardiovascular health and diseases in china, 2022; Tokgozoglu et al., 2021). The COVID-19 pandemic has increased the burden of cardiovascular disease, as studies indicate that heart patients are at higher risk of serious complications (Tolu-Akinnawo et al., 2023). Heart failure is primarily caused by coronary artery disease and myocardial ischemia, which often occur simultaneously (Roberts et al., 2023). Conventional treatments for cardiovascular disease include catheter-based procedures such as angioplasty to open blocked arteries and surgical procedures such as coronary artery bypass grafts or organ transplants for end-stage heart failure. These treatments may also include the prescription of cardioprotective medications such as beta blockers, calcium channel blockers, or oral diuretics (Zhu et al., 2023; Loosen et al., 2022). While these cardioprotective therapies have been shown to improve cardiac function in patients with coronary artery disease and prevent adverse cardiac events after an initial episode of cardiac arrest, their long-term benefits in disease recovery are marginal. Furthermore, their continued use may be associated with serious side effects that may overshadow their positive effects on patients. Nevertheless, these treatments are not sufficient to regenerate or repair the cardiac environment, limiting their role in cardiac repair (Khalpey et al., 2024; Cortese et al., 2023; Krittanawong et al., 2024).

**FIGURE 1:**
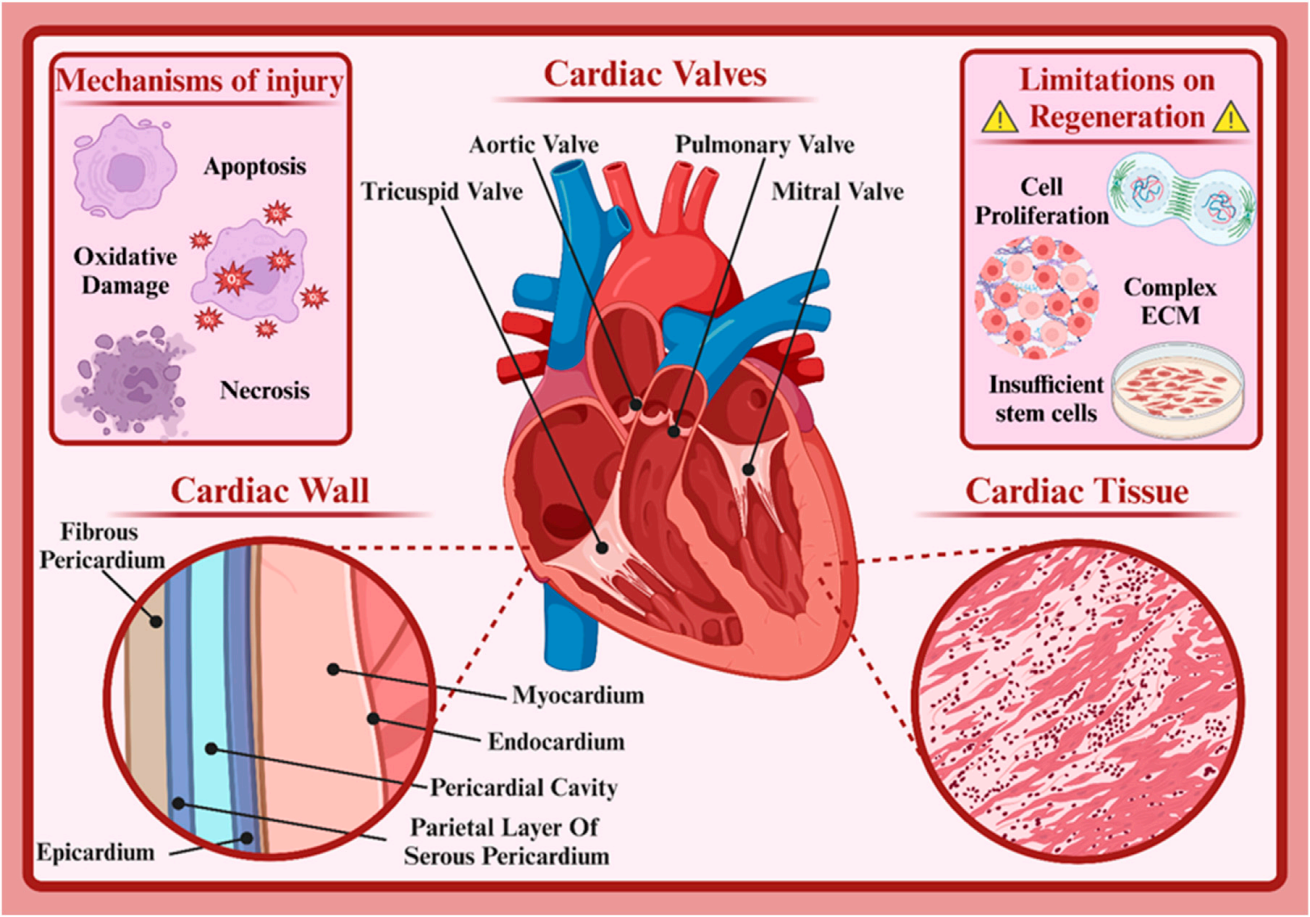
Cardiac structure. Created with BioRender.com.

The human heart serves as the muscular pump that circulates blood throughout the body, delivering oxygen and nutrients to tissues. This vital structure consists of four chambers, the left and right atria and the left and right ventricles (Iacobas et al., 2021). The left atrium receives oxygenated blood from the lungs, and deoxygenated blood from the rest of the body enters the right atrium (Kadiyala et al., 2021). Additionally, the left ventricle pumps oxygenated blood from the heart throughout the body, and the right ventricle pumps deoxygenated blood to the lungs (Kadiyala et al., 2021). Four valves (the mitral, aortic, tricuspid, and pulmonary valves) regulate blood flow between the chambers, preventing backflow and ensuring unidirectional circulation (Figure 1) (Yan S. et al., 2022). The heart’s electrical conduction system generates and conducts electrical signals that coordinate the heart’s contractions. This system includes the sinoatrial (SA) node, the atrioventricular (AV) node, and the bundle of His (Nissen et al., 2022; van der Maarel and Christoffels, 2024). The SA node, often referred to as the heart’s pacemaker, initiates electrical impulses that are then propagated throughout the heart, causing it to contract rhythmically (van der Maarel and Christoffels, 2024).

The heart is composed of various types of cells, each playing a specific role in its function. Understanding these cardiac cell types and their functions aids physicians in better diagnosing and treating heart diseases (Santos et al., 2021; Yao et al., 2024). The specialized muscle cells responsible for the heart’s contractions are called cardiomyocytes. These long, branched cells are connected by intercalated discs that facilitate rapid electrical impulse transmission between cells, ensuring coordinated contractions (Billur et al., 2023; Struckman et al., 2023). There are two types: atrial and ventricular cardiomyocytes, differing in phenotype and function. Atrial cardiomyocytes, located in the atrial walls, initiate and conduct electrical impulses within the heart. The sinoatrial (SA) node, the heart’s natural pacemaker, is composed of these cells (Easterling et al., 2021). Ventricular cardiomyocytes, found in the ventricular walls, are responsible for forceful contractions. Their thick muscle fibers enable powerful contractions (Bruns et al., 2024). Cardiac fibroblasts are connective tissue cells producing the heart’s extracellular matrix. This matrix provides structural support, anchoring cardiac cells and facilitating cell-to-cell communication. It also plays a role in heart tissue repair post-injury (Guilak et al., 2021; Micheletti and Alexanian, 2022; Phogat et al., 2023). Neural cells in the heart regulate heart rate and its response to physiological changes. They receive neural signals from the autonomic nervous system and transmit them to cardiac cells (Yu Y. et al., 2023). Endothelial cells line the inner layer of the heart’s blood vessels. They play crucial roles in regulating blood flow, exchanging substances between blood and heart tissue, and in inflammatory responses (Brandt et al., 2022; Ribatti, 2024). Smooth muscle cells are found in the walls of heart blood vessels and other cardiac structures. They regulate blood vessel diameter, thus controlling blood flow to the heart (Hernandez-Hernandez et al., 2024). Each type of cardiac cell is vital to the heart’s function. Dysfunction of any of these cells can lead to heart diseases. For instance, cardiomyocyte damage can result in heart failure, while fibroblast dysfunction can lead to cardiac fibrosis (Levick and Widiapradja, 2020).

Numerous factors such as neural factors, hormones, mineral ions, drugs, and diseases affect the heart’s function. Damage to cardiac cells, or cardiomyocytes, is one of the most important causes of cardiovascular diseases. This damage can occur due to various reasons, including coronary artery disease, high blood pressure, diabetes, infections, and genetic factors (Ahn et al., 2023; Arjmand et al., 2021). Damage to cardiac cells usually occurs due to reduced blood flow to the heart muscle. This reduced blood flow can be caused by coronary artery blockage, vascular spasm, or decreased blood pressure. As a result of reduced blood flow, cardiac cells are deprived of oxygen and nutrients and are susceptible to damage. Cardiac cell damage can be classified into reversible and irreversible damage (Fan et al., 2023; Jenkins et al., 2024). In reversible damage, cardiac cells are still alive but their function is temporarily impaired. With the removal of the causative agent, cells can return to their normal state. In irreversible damage, cardiac cells are completely destroyed and cannot be replaced. This type of damage leads to the formation of scar tissue in the heart and can reduce the heart’s pumping function (Smagul et al., 2020).

Various processes occur that lead to cell damage. Reduced oxygen supply to cells causes the production of free radicals that damage cellular components (oxidative damage). Damaged cells undergo programmed cell death (apoptosis) to prevent further damage to the tissue. Also, in cases of severe damage, cells die suddenly (necrosis) and cause inflammation and release of harmful substances into the surrounding environment (Chen J. et al., 2023; Song et al., 2022; Wu et al., 2020). The heart has a very limited ability to repair itself after injury. These limitations are due to various reasons, including the inherent characteristics of cardiac cells and the complexity of heart tissue, which will be discussed below (Lin et al., 2020; Tran et al., 2023).

Adult cardiac cells typically do not have the ability to divide and proliferate, which means that after injury, the number of new cells produced to replace damaged cells is very limited (Sadahiro and Ieda, 2022; Zuppo et al., 2023). Cardiac cells are arrested in the G1 phase of the cell cycle and therefore cannot easily enter the cell cycle and divide (Dorr et al., 2015). The extracellular matrix of the heart has a very stiff and complex structure, which limits the migration and proliferation of new cells and makes it difficult to create new blood vessels and form new tissue (Tu et al., 2023). On the other hand, after heart injury, a chronic inflammatory response occurs that can damage healthy cardiac cells, disrupt the repair process, and lead to the formation of scar tissue, which prevents the regrowth of healthy heart tissue (Sun K. et al., 2021). Also, the number of cardiac stem cells is very limited, and these cells have a limited ability to produce new cardiac cells (Femmino et al., 2022). Finally, the heart is composed of various types of cells, each with a specific role. Replacing these cells with new cells in a precise and coordinated manner is very difficult (Scemama et al., 2023). The limitations of heart repair are a major challenge in medicine. However, extensive research is underway in the fields of stem cells, tissue engineering, and gene therapy to find new ways to repair damaged heart tissue.

The amniotic membrane (AM) is a thin biological layer that protects the developing fetus in the mother’s womb. It is rich in proteins, growth factors, cytokines, and other biomolecules that play an important role in preventing scar formation, reducing inflammation, and promoting tissue regeneration. When AM comes into contact with cardiac tissue, specific cellular interactions occur between them that promote various cellular behaviors such as maturation, proliferation, and migration, as well as the activation of specific signaling pathways that facilitate the process of repairing damaged tissue. In addition, AM, despite its excellent flexibility, also offers significant strength that can be used in stress-related applications. Therefore, AM can be used to develop many bioscaffolds in the field of tissue regeneration, especially cardiac tissue (Sarvari et al., 2022; Zamproni et al., 2021). In this review article, we will explore the applications of amniotic membrane in treating cardiovascular diseases. We will begin by introducing the structure and biological properties of the amniotic membrane and then examine the mechanisms of action of this membrane in repairing cardiac and vascular tissues. Subsequently, we will review various studies conducted in this field and specifically focus on the applications of amniotic membrane in treating ischemic heart disease, atherosclerosis, and heart failure. Finally, we will address the challenges and limitations of using amniotic membrane in this field and the future prospects of this area.

## 2 Amniotic membrane

The amniotic membrane (AM), commonly referred to as the “true embryonic membrane,” is composed of an epithelium, a basement membrane (BM), and a thick, dense stroma (Doudi et al., 2022). Due to this unique cell composition, AM is a complex tissue with exceptional properties such as immunogenicity, anti-inflammatory effects and high antibacterial activity, among many others (Canciello et al., 2020; Gholipourmalekabadi et al., 2020; Kafili et al., 2024; Law et al., 2022). Ioannis Postonce was the first to describe the AM and its function in 1910. He hypothesized that if AM has the function of protecting the developing fetus from any changes in the uterine environment, AM could potentially help preserve the cellular properties of differentiated cells (Lanci et al., 2022; Ramuta et al., 2020). With advances in medical science and more in-depth studies, scientists have discovered the amazing properties of the amniotic membrane. This membrane is rich in growth factors, cytokines, and other biomolecules that play critical roles in tissue repair, reducing inflammation, and preventing scar formation. In the following sections, we will examine the formation stages, structure, and biological properties of AM.

### 2.1 Amniotic membrane formation

After an ovum is fertilized by a sperm, the zygote divides rapidly to form a mass of cells called a blastocyst. The blastocyst consists of two main parts: the inner cell mass and the outer cell mass (Nguyen et al., 2024). The inner cell mass develops into the embryo, while the outer cell mass develops into the placenta and embryonic membranes. During the early stages of embryonic development, the blastocyst, a hollow ball-like structure, undergoes significant morphological changes (Martinez de Los Reyes et al., 2024). The outer cell mass of the blastocyst, known as the trophoblast, differentiates into two distinct layers: the epiblast and the hypoblast. These two layers play a key role in the formation of primary embryonic structures. The epiblast, a columnar layer of cells, faces the blastocyst cavity (Harmoush et al., 2021). The epiblast undergoes an inward folding process called gastrulation. This folding leads to the formation of a new cavity, the amniotic cavity. The epiblastic cells that surround this cavity are called amnioblasts. They multiply gradually and form a thin membrane that lines the amniotic cavity. This membrane is called the amniotic membrane and is where the embryo ultimately develops (Harmoush et al., 2021). Simultaneously with the formation of the amniotic cavity, the hypoblast cells also fold inward, forming another cavity called the primary yolk sac. The primary yolk sac plays an important role in early embryonic development, serving as a site for blood cell formation and nutrient transfer to the embryo (Hislop et al., 2023; Ornoy and Miller, 2023).

Amnioblasts multiply rapidly and form a simple epithelial layer on the inner surface of the amniotic cavity. This epithelial layer forms the amniotic membrane. As pregnancy progresses, the amniotic membrane enlarges along with the growing fetus, completely lining the amniotic cavity. As the number of cell layers increases and the secretion of extracellular matrix by amnioblasts, the amniotic membrane becomes thicker and more robust (Fitriani et al., 2023; Kim et al., 2024).

Amnioblast cells actively secrete amniotic fluid. This fluid provides an aqueous and sterile environment for the fetus and performs important functions such as mechanical protection, regulating fetal temperature, facilitating fetal movement, and promoting lung development (Shamsnajafabadi and Soheili, 2022). Amniotic fluid consists primarily of water, proteins, lipids, glucose, and minerals. As the volume of amniotic fluid increases, the amniotic cavity enlarges, allowing the fetus to float freely. In addition to the amniotic membrane, other embryonic membranes also form. These include chorion and allantois. The chorion is the outermost embryonic membrane and separates the fetus from the uterine wall. The allantois also plays an important role in the formation of the umbilical cord and fetal blood vessels (Zhu et al., 2022).

### 2.2 Structure of the amniotic membrane

**FIGURE 2:**
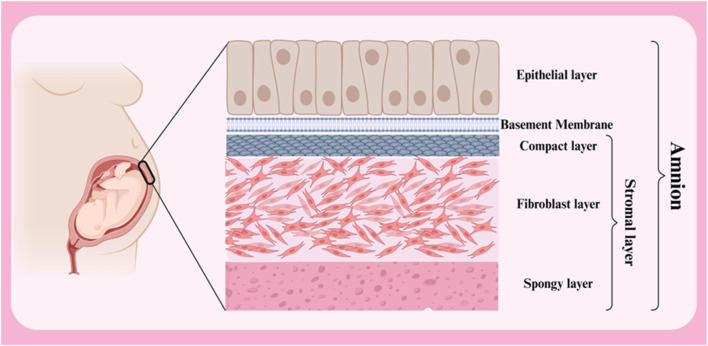
Different layers of amnion membrane. Created with BioRender.com.

The amniotic membrane is a thin, transparent biological structure that encases the developing fetus in the mother’s uterus. This membrane is extremely flexible and allows the fetus to grow. Despite its thinness, the amniotic membrane has sufficient strength to protect the fetus from external shocks. In addition, it is avascular, meaning it has no blood vessels and receives its necessary nutrients through diffusion from the amniotic fluid. The amniotic membrane has antimicrobial properties and acts as a barrier against the penetration of microbes into the amniotic cavity. In general, the amniotic membrane consists of three primary layers (from outside to inside: epithelial layer, basement membrane and stromal layer) (Figure 2) (Table 1) (Doudi et al., 2022; Shamsnajafabadi and Soheili, 2022). Below is a detailed discussion of each of these layers.

**TABLE 1:** Substances and factors secreted from each layer of the amniotic membrane.

Layer	Main secreted materials and factors	Main role	Ref
Epithelial	Albumin, immunoglobulins, growth factors (EGF, FGF, TGF-β), lipids (phospholipids, cholesterol), carbohydrates (glucose, glycogen), ions (sodium, potassium, calcium), cytokines (interleukins, TNF)	Maintaining amniotic fluid homeostasis, fetal nutrition, fetal protection, lung development	Bertolin et al. (2022b), Ghamari et al. (2020), Iizuka et al. (2019)
Basement membrane	Growth factors (laminin, entactin, fibronectin), cell adhesion molecules	Attachment of epithelial cells, filtration of materials, cell signaling	Iranpour et al. (2018), Kolundzic et al. (2022), Shariatzadeh et al. (2021)
Stromal	Growth factors (EGF, FGF, TGF-β), cytokines (interleukins, TNF), collagen, elastin, proteoglycans	Wound healing, reducing inflammation, formation of new tissue, regulating immune response	Tauseef et al. (2024), Ghamari et al. (2020), Bhatti et al. (2023), Lin et al. (2023)

#### 2.2.1 Epithelial layer

Epithelial Layer is the outermost layer of the membrane and consists of a single layer of simple squamous cells. These cells play a crucial role in protecting the fetus and creating a sterile environment for its growth. These cells are arranged regularly and compactly and form a continuous shell. The amniotic fluid epithelial cells have a pronounced polarity, with the apical part of the cells facing the amniotic cavity and covered with short microvilli, while the basal part is attached to the basement membrane (Kim et al., 2024). Amniotic fluid epithelial cells are connected to each other by tight junctions and desmosomes, which prevent pollutants and microorganisms from entering the amniotic cavity. In addition, these cells secrete various substances such as growth factors, proteins and lipids, which play a role in maintaining amniotic fluid homeostasis and fetal growth (Wu et al., 2019).

This layer provides physical protection to the fetus by acting as a protective barrier against pathogens, harmful chemicals, and mechanical forces, while selectively allowing the passage of certain substances and preventing the passage of harmful materials. This layer plays a crucial role in maintaining amniotic fluid homeostasis. Epithelial cells regulate the composition of amniotic fluid by secreting various substances such as proteins, ions, growth factors and more. In addition, this layer facilitates the transfer of certain nutrients from the amniotic fluid to the fetus via the epithelial layer (Han et al., 2021; Kawamura et al., 2022).

#### 2.2.2 Basement membrane

Basement Membrane is a thin, dense layer of collagen and other proteins. The basement membrane plays a crucial role in attaching the epithelial layer to the stromal layer (maintaining the structural integrity of the membrane) and also acts as a filtration barrier. The basement membrane is mainly composed of a complex network of structural proteins, particularly collagen types IV, V and VII. These proteins, together with other non-collagenous proteins such as laminin, entactin and fibronectin, form a strong and flexible extracellular matrix. This matrix is organized as a lattice-like layer and serves as a scaffold for the attachment of epithelial cells. By binding to receptors on the basal surface of epithelial cells, the basement membrane firmly anchors these cells in place. This binding is important for maintaining the integrity of the epithelial layer and preventing cells from separating from one another. This membrane acts as a filter and regulates the passage of substances between the different layers of the amniotic membrane. The pore size and protein composition of the basement membrane determine the type of substances that can pass through it. The basement membrane contains signaling molecules that play a role in regulating the growth, proliferation, and differentiation of neighboring cells. These molecules can influence gene expression and cellular activities. Additionally, this membrane creates a specialized microenvironment for adjacent cells, allowing them to perform their specialized functions (Doudi et al., 2022; Bertolin et al., 2022a; Chen et al., 2024).

The basement membrane plays a fundamental role in maintaining the health of the fetus and the proper functioning of the amniotic membrane. Any disruption in the structure or composition of the basement membrane can lead to a variety of problems, including impaired fetal growth, leakage of amniotic fluid, and an increased risk of infection. In summary, the amniotic basement membrane is a complex and multifunctional structure that plays a crucial role in maintaining the integrity of the amniotic membrane and regulating material exchange (Tauseef et al., 2024).

#### 2.2.3 Stromal layer

This is the innermost layer of the membrane and consists of loose connective tissue. This layer contains various cells, including fibroblasts, macrophages and mesenchymal stem cells, which play a crucial role in the production of growth factors, cytokines and other biomolecules that give the amniotic membrane its therapeutic properties. This layer acts as a dynamic matrix and plays an important role in regulating tissue repair and regeneration processes. The stromal layer is composed of various cell types, including fibroblasts, macrophages, and mesenchymal stem cells, each playing a specific role in the function of this layer. Fibroblasts in this layer are responsible for producing the extracellular matrix, which consists of collagen, elastin and proteoglycans. The extracellular matrix serves as a scaffold for other cells and gives them shape and support. Macrophages form the innate immune system and are activated in response to injury or infection. They help protect tissue by engulfing pathogens and producing cytokines. Mesenchymal stem cells have the ability to differentiate into different types of connective tissue cells. Mesenchymal stem cells play a crucial role in wound healing and tissue regeneration (Fitriani et al., 2023; Weidinger et al., 2020).

Due to the presence of various types of cells and the production of a wide range of biomolecules, the stromal layer gives the amniotic membrane unique therapeutic properties:

• Production of growth factors: Cells in the stromal layer produce various types of growth factors that play a role in stimulating cell growth, tissue repair, and reducing inflammation. Examples of these factors include epidermal growth factor (EGF), fibroblast growth factor (FGF), and transforming growth factor beta (TGF-β) (Pogozhykh et al., 2020).
• Production of cytokines: Cells in the stromal layer produce cytokines that regulate the immune response and inflammation. The anti-inflammatory cytokines produced by these cells help to reduce inflammation and accelerate the healing process (Cervero-Varona et al., 2023; Correa et al., 2022).
• Wound healing: Mesenchymal stem cells present in the stromal layer can migrate to the site of injury and, by differentiating into the cells of the tissue in question, help to repair the wound (Messmer, 2020; Permkam et al., 2022).
• Reduced scar formation: Some of the molecules produced by the cells of the stromal layer can help to reduce scar formation (Messmer, 2020; Permkam et al., 2022).

### 2.3 Properties of amniotic membrane

The amniotic membrane not only acts as a protector for the fetus but also possesses valuable therapeutic properties. This membrane contains a wide range of growth factors, cytokines, and extracellular matrix, which play a significant role in tissue repair, inflammation reduction, and immune response regulation (Fitriani et al., 2023; Jahanafrooz et al., 2023). The biological properties of the amniotic membrane have made it an attractive option for applications in regenerative medicine (Table 2). This membrane can be used as a biological scaffold for repairing damaged tissues, reducing inflammation, and accelerating the healing process (Etchebarne et al., 2021). In the following, we will examine some of the important properties of this membrane.

**TABLE 2:** Biological properties of amniotic membrane.

Property	Description	Mechanism of action	Key factors	Clinical applications	Ref
Anti-inflammatory	Inhibits inflammatory response, reduces production of pro-inflammatory cytokines (TNF-α, IL-1β) and increases anti-inflammatory cytokines (IL-10, TGF-β)	Interacts with cellular receptors, inhibits inflammatory signaling pathways, creates an anti-inflammatory environment	Growth factors (EGF, FGF, TGF-β), proteoglycans, mesenchymal stem cells	Chronic wound healing, burns, orthopedic surgeries, autoimmune diseases	Messmer (2020), Manti et al. (2022), Lan et al. (2020), Duerr et al. (2019), Gera et al. (2023), Karaca et al. (2024), Valiente et al. (2018)
Antimicrobial	Inhibits growth of bacteria, viruses, and some fungi	Presence of antimicrobial peptides, creation of acidic environment, formation of physical barrier	Antimicrobial peptides, low pH, fibrous structure	Prevention of wound infection, wound dressings, surgeries	Tauseef et al. (2024), Maekawa et al. (2021), Hisey et al. (2023), Bulut et al. (2023), Lohajaroensub et al. (2022), Sket et al. (2021), Syed and Rapuano (2021), Ting et al. (2020), Wali et al. (2022)
Immunomodulatory	Regulates immune response, reduces excessive immune response	Interacts with immune cells (macrophages, lymphocytes), regulates expression of adhesion molecules	Mesenchymal stem cells, growth factors, extracellular matrix	Organ transplantation, autoimmune diseases, allergies	Canciello et al. (2020), Tauseef et al. (2024), Abou-Shanab et al. (2023), Alonso-Carpio et al. (2020), Canciello et al. (2024), de Oliveira Pinheiro et al. (2020), Riedel et al. (2023), Skowron-Kandzia et al. (2021), Wassmer and Berishvili (2020)
Anti-scarring	Reduces scar tissue formation, promotes natural tissue repair	Regulates collagen synthesis, inhibits myofibroblast activity	Growth factors (TGF-β), extracellular matrix, mesenchymal stem cells	Burn treatment, surgical wounds, cosmetic surgery	Law et al. (2022), Gera et al. (2023), Hazarika et al. (2022), Li et al. (2023b), Munoz-Torres et al. (2022), Shabani et al. (2020)
Tissue regenerative	Stimulates cell proliferation and migration, promotes new blood vessel formation (angiogenesis), synthesizes extracellular matrix	Presence of growth factors (VEGF, FGF), extracellular matrix, mesenchymal stem cells	Growth factors, extracellular matrix, mesenchymal stem cells	Tissue engineering, chronic wound healing, treatment of ischemic diseases	Etchebarne et al. (2021), Wassmer and Berishvili (2020), Dasargyri et al. (2023b), Elkhenany et al. (2022), Gupta, 2022; Hu et al. (2023), Ingraldi et al. (2023), Mirzadegan et al. (2020)

#### 2.3.1 Mechanical properties

As a biological tissue, the amniotic membrane has various mechanical properties that play an essential role in its function and therapeutic applications. These mechanical properties are influenced by various factors, including fetal age, location of membrane collection, and processing methods (Hu et al., 2024). One of the most notable properties of the amniotic membrane is its high flexibility and extensibility, which allows the membrane to adapt to changes in the volume and shape of the amniotic cavity during pregnancy and to withstand tensile forces without rupturing (Iizuka et al., 2019; Ikeda et al., 2023). Tensile strength is determined by the alignment of collagen fibers in the extracellular matrix (ECM), while elastic deformation is associated with the presence of elastin fibers, laminin, hyaluronic acid, and glycosaminoglycans (Bennasroune et al., 2019; Runci Anastasi et al., 2021). The amniotic membrane has significant tear resistance, which helps protect the fetus from external mechanical forces (Koh et al., 2019). The tensile strength depends on the thickness, the collagen density, and the arrangement of the collagen fibers within the extracellular matrix (Gao et al., 2022).

#### 2.3.2 Biodegradation

The amniotic membrane, due to its biological nature and predominant collagen composition, undergoes natural biodegradation processes. During this process, the tissue is broken down by certain cells and natural enzymes (Sarvari et al., 2022). This process is influenced by various factors, including tissue composition (collagen concentration, presence of other proteins and minerals), environmental conditions (temperature, humidity, and pH), and the type of microorganisms present in the environment (different species of microorganisms produce different enzymes and attack the tissue at varying rates) (Lu et al., 2024; Mizutani et al., 2022). Processing methods such as sterilization and drying also affect the tissue structure and consequently the rate of biodegradation. The degradation of hAM can vary from a few days to several months (depending on the species and site of application/implantation), however, the data is not well documented (Nasiry et al., 2021; Odet et al., 2022; Peng et al., 2023).

Mechanisms such as hydrolysis, oxidation, and biofilm formation influence the biodegradation of the amniotic membrane (Maekawa et al., 2021; Mandal et al., 2017; Sanchez-Huerta et al., 2023). Peptide bonds in collagen molecules are broken down by proteases produced by microorganisms, leading to the degradation of collagen into smaller peptides and amino acids (Kviatkovsky et al., 2022). On the other hand, oxygen free radicals and oxidase enzymes can attack the side chains of amino acids in collagen, causing protein structure damage (Carretero et al., 2023).

#### 2.3.3 Cellular compatibility

The amniotic membrane, as a unique biological scaffold, has found widespread applications in tissue engineering and regenerative medicine. One of the primary reasons for these applications is its exceptional ability to facilitate cell adhesion, proliferation, and differentiation. These properties depend on various factors, including the composition of the extracellular matrix (ECM), the presence of growth factors and cytokines, and its microscopic structure (Tauseef et al., 2024; Becker et al., 2018).

Cell adhesion is a vital process in which cells attach to each other or to the extracellular matrix (ECM) (Ryu et al., 2022). This process is essential for tissue formation, healing, and many other biological processes. The amniotic membrane, due to its unique ECM composition, plays a significant role in facilitating cell adhesion (Barski et al., 2015; McDaniel et al., 2021). The amniotic membrane is rich in proteins such as fibronectin, laminin, collagen, and vitronectin. These proteins act as ligands and bind to specific receptors on the cell surface, such as integrins (Ahmed et al., 2024; Ueda et al., 2022). Integrins are transmembrane proteins that bind to adhesive proteins in the ECM (Yu C. et al., 2023). This binding sends signals into the cell that lead to changes in the cytoskeleton and the formation of adhesive structures such as focal adhesions (Iwamoto and Calderwood, 2015). In addition to integrins, other adhesion molecules such as cadherins and selectins also play a role in cell adhesion (Kim et al., 2020; Zinellu and Mangoni, 2024).

Cell proliferation is a fundamental process in tissue growth and repair (Dommann et al., 2022). In tissue engineering and regenerative medicine, the ability of a biological scaffold to facilitate cell proliferation is a key factor in the success of treatment (Trubiani et al., 2019). The amniotic membrane, as a natural biological scaffold, has gained significant attention in this field due to its specific composition and microenvironment that supports cell proliferation (Iranpour et al., 2018; Li et al., 2023a). Proteins such as fibronectin, laminin, and collagen in the amniotic membrane ECM act as receptors for cells, promoting their adhesion to the scaffold. This adhesion sends signals to cells that activate signaling pathways and ultimately lead to cell proliferation (Shen et al., 2019). Additionally, proteoglycans, by absorbing water, provide a moist and nutrient-rich environment for cells, thus supporting their proliferation (Hannon et al., 2019). Growth factors such as EGF, FGF, and TGF-β stimulate the proliferation of epithelial cells, fibroblasts, endothelial cells, and mesenchymal stem cells (Wu et al., 2017). Furthermore, cytokines such as interleukins (IL) and tumor necrosis factor (TNF) can influence cell proliferation and regulate the inflammatory response (Souza et al., 2023).

#### 2.3.4 Anti-inflammatory

Amniotic membrane possesses inherent anti-inflammatory properties, containing factors that suppress the inflammatory response and facilitate expedited healing. The underlying mechanisms of its anti-inflammatory action are multifaceted (Manti et al., 2022).

One significant mechanism involves the inhibition of pro-inflammatory cytokines. The amniotic membrane contains factors that suppress the production and release of pro-inflammatory cytokines such as TNF-α, IL-1β, and IL-6. These cytokines play a pivotal role in the inflammatory response, and reducing their levels contributes to a decrease in inflammation (Kirici et al., 2022; Wang C. et al., 2024). Conversely, this membrane also stimulates the production of anti-inflammatory cytokines like IL-10 and TGF-β, which exhibit potent anti-inflammatory effects and promote tissue repair (Li et al., 2022). Furthermore, the amniotic membrane stabilizes cell membrane structure, reducing lipid peroxidation. This prevents membrane damage, subsequent release of intracellular contents (such as lysosomal contents), and the inappropriate activation of pattern recognition receptors (PRRs), thereby mitigating the inflammatory response. Certain components of the amniotic membrane can absorb proteolytic enzymes involved in the inflammatory process, further contributing to reduced inflammation (von Krusenstiern et al., 2023). By modulating the immune response, the amniotic membrane fosters a balanced inflammatory environment and protects damaged tissues. Through mechanisms such as regulating the expression of cell adhesion molecules, modulating the production of cytokines and chemokines, and influencing the function of both innate and adaptive immune cells, it prevents an excessive immune response that could lead to tissue damage (Ramachandran et al., 2024).

#### 2.3.5 Antimicrobial

Numerous studies have demonstrated that the amniotic membrane possesses a broad spectrum of antimicrobial properties, including activity against bacteria, viruses, and certain fungi. The mechanism underlying this antimicrobial effect can be attributed to various factors such as the presence of antimicrobial peptides, changes in the pH environment, and the creation of a physical barrier (Kumaresan et al., 2024).

The amniotic membrane contains a wide array of natural antimicrobial peptides (AMPs) that directly target the cell membranes of microorganisms, causing their destruction. These peptides employ diverse mechanisms of action, including pore formation in cell membranes, disruption of protein and nucleic acid synthesis, and activation of the innate immune system (Hisey et al., 2023; Moosazadeh Moghaddam et al., 2023). Moreover, the environment surrounding the amniotic membrane typically exhibits an acidic pH, which is detrimental to many microorganisms and inhibits their growth. This acidic pH can directly affect the cell membranes of microorganisms, impairing their function. It is important to note that certain microorganisms, such as some bacteria (like Group B *Streptococcus*, *Listeria* monocytogenes, and certain species of *Klebsiella*), some viruses (like Zika virus, rubella virus, and certain influenza viruses), and some fungi (like *Candida* albicans) can penetrate the amniotic membrane (Johnston et al., 2024; Masumoto et al., 2024; Sani et al., 2023; Yan P. et al., 2022).

#### 2.3.6 Modulation of the immune response

The amniotic membrane employs various mechanisms to prevent an excessive immune response and maintain immune homeostasis. The most significant mechanisms that modulate the immune response are discussed below:

• Reduced production of pro-inflammatory cytokines: The amniotic membrane decreases the production of pro-inflammatory cytokines such as TNF-α, IL-1β, and IL-6, thereby reducing the severity of the inflammatory response. These cytokines play a crucial role in activating the immune system and inducing inflammation (Wang et al., 2023; Wei et al., 2023).
• Increased production of anti-inflammatory cytokines: This membrane stimulates the production of anti-inflammatory cytokines like IL-10 and TGF-β. These cytokines inhibit the activity of immune cells and decrease the production of inflammatory molecules, contributing to a reduction in inflammation (Wang et al., 2023; Evangelista et al., 2021).
• Modulation of immune cell activity: The amniotic membrane influences the activity of immune cells such as macrophages, neutrophils, and lymphocytes, helping to regulate the immune response. This membrane can inhibit the activity of some of these cells while stimulating the activity of others. The amniotic membrane also has low antigenicity and rarely triggers an immune rejection reaction. This feature is due to the low expression of MHC class I and II molecules in amniotic epithelial cells (Gomez-Lopez et al., 2019; Xu et al., 2024; Dasargyri et al., 2023a).
• Creation of an anti-inflammatory environment: By absorbing proteolytic enzymes and free radicals, the amniotic membrane creates an anti-inflammatory environment. This environment prevents damage to healthy tissues and promotes tissue repair (Alfoldi and Ferianec, 2024; Liu et al., 2023; Antoine et al., 2022).
• Stabilization of the cell membrane: By stabilizing the cell membrane, the amniotic membrane prevents the release of intracellular contents and the activation of the inflammatory response (Tauseef et al., 2024; Lan et al., 2020).

#### 2.3.7 Anti-scarring

In addition to its anti-inflammatory and antimicrobial properties, the amniotic membrane also exhibits anti-scarring effects. This means that the amniotic membrane can prevent the excessive formation of scar tissue and promote the healing of natural tissue. This property is influenced by factors such as the inhibition of type III collagen synthesis, the regulation of growth factors, the modulation of the immune response, the provision of a moist environment, and the reduction of the inflammatory response (Liu et al., 2023; Lin-Hui et al., 2024).

Scar tissue is primarily composed of type III collagen. By inhibiting the synthesis of type III collagen and stimulating the synthesis of type I collagen, the amniotic membrane contributes to improved tissue quality and reduced scarring. Type I collagen is stronger and more flexible than type III collagen and gives the tissue a more natural appearance (Lin-Hui et al., 2024). Additionally, the amniotic membrane contains various growth factors such as TGF-β, PDGF, and VEGF (Liu C. et al., 2020). These factors play a crucial role in regulating the wound healing process and preventing excessive scar formation. Furthermore, by creating a moist environment, the amniotic membrane promotes cell migration and the formation of new tissue. Given its unique natural properties, the amniotic membrane has become an ideal option in tissue engineering and cell therapy research. This membrane is used in treatments such as ocular diseases, skin diseases, ischemic diseases, and more (Chen L. et al., 2023; Erkoc-Biradli et al., 2024).

## 3 Effects of amniotic membrane on damaged heart cells

Given its unique biological properties, the amniotic membrane has emerged as a promising bio-scaffold in regenerative medicine, particularly in cardiac tissue repair. When the amniotic membrane is in contact with cardiac cells, interactions occur between them. A deep understanding of these cellular interaction mechanisms can contribute to the development of new therapeutic strategies for heart diseases. For instance, this knowledge can be used to design novel biomaterials that can serve as scaffolds for cardiac tissue repair. Additionally, it can be utilized to engineer cardiac stem cells to produce new cardiac cells to replace damaged ones. In this section, we will delve into the detailed cellular interactions between the amniotic membrane and damaged cardiac cells. Cellular interactions are complex processes that play a role in many vital bodily functions including growth, tissue repair, and immune response. One of the most important of these interactions is cell adhesion, which allows cells to adhere to each other or to the extracellular matrix. This adhesion, in addition to maintaining the structure of tissues, is also essential for cell migration. The amniotic membrane contains proteins such as fibronectin, laminin, and collagen that act as cell adhesion molecules (Munoz-Torres et al., 2022). These molecules have specific binding sites that attach to receptors on the surface of cardiac cells. This binding leads to the formation of strong bonds between cardiac cells and the amniotic membrane. This adhesion provides a stable and supportive environment for cardiac cells, aiding in their growth and proliferation (Graf et al., 2021; Hsieh et al., 2023; Kiyozumi et al., 2020). Cardiac cells adhering to the amniotic membrane can migrate in a directed and organized manner along the surface of the membrane, which is essential for the repair of damaged cardiac tissues. Furthermore, the interaction between cardiac cells and the amniotic membrane can regulate various cellular activities such as gene expression, protein production, and cell signaling (Connell et al., 2015). The molecular mechanisms underlying these interactions are very complex and multi-step. In summary, this process involves recognition, binding, formation of adhesion complexes, and regulation of the cytoskeleton (Li et al., 2016; Revach et al., 2020). The receptors on the surface of cardiac cells recognize the cell adhesion molecules present in the amniotic membrane. After recognition, the receptors bind to the cell adhesion molecules, forming initial bonds. These bonds are then gradually strengthened, forming complex adhesion complexes. Ultimately, this leads to changes in the organization of the cell cytoskeleton, which in turn affects the cell’s shape, movement, and adhesion (Connell et al., 2015).

In addition to cell adhesion molecules, the amniotic membrane contains a wide range of growth factors that play a vital role in the process of cardiac tissue repair. These growth factors include VEGF (Vascular Endothelial Growth Factor), FGF (Fibroblast Growth Factor), and TGF-β (Transforming Growth Factor beta) (Liu Z. et al., 2020; Oba et al., 2020). The growth factors present in the amniotic membrane interact with specific receptors on the surface of cardiac cells. These receptors are transmembrane proteins specifically designed to recognize and bind to a particular growth factor. Many of these growth factors present in the amniotic membrane interact with receptors called receptor tyrosine kinases (RTKs), which have a specific structure that includes an extracellular domain, a transmembrane domain, and an intracellular domain (Trenker and Jura, 2020). The extracellular domain contains specific binding sites for growth factors, and when a growth factor binds to this site, two receptor molecules come together and form a dimer (Nielsen et al., 2022). The intracellular domain has kinase activity, and after the dimerization of the receptors, the intracellular domain is activated and begins to phosphorylate tyrosine residues in itself and other proteins (Albrecht et al., 2020). Protein phosphorylation initiates a chain of biochemical reactions, and these pathways transmit information from the extracellular environment to the cell nucleus, ultimately leading to changes in gene expression and cellular activities (Kagiwada et al., 2021).

The activation of intracellular signaling pathways results in a wide range of biological effects that are essential for cardiac tissue repair, such as stimulating cell proliferation, cell migration, extracellular matrix synthesis, and angiogenesis. Growth factors stimulate cell division and increase the number of cardiac cells. These factors guide cardiac cells toward the site of injury and facilitate their migration. Growth factors stimulate the synthesis of extracellular matrix proteins such as collagen and elastin, which are essential for the regeneration of damaged tissue (Guerreiro et al., 2021; Vu et al., 2022). VEGF, in particular, acts on vascular endothelial cells, promoting the formation of new blood vessels, a process that is essential for supplying the oxygen and nutrients required for tissue repair (Hida et al., 2001; Testa et al., 2020). In summary, Table 3 presents the growth factors present in the amniotic membrane and their effects on cardiac cells.

**TABLE 3:** The growth factors present in the amniotic membrane and their effects on cardiac cells.

Growth factor	Primary effect on cardiac cells	Mechanism of action	Ref
VEGF (Vascular Endothelial Growth Factor)	Promotes angiogenesis, increases vascular permeability, promotes proliferation of endothelial cells	Activates tyrosine kinase receptors (VEGFR) and stimulates PI3K/Akt and MAPK pathways	Koizumi et al. (2022), Mariotti et al. (2021), Wang et al. (2024b), Wang et al. (2020)
FGF (Fibroblast Growth Factor)	Promotes cell proliferation, cell migration, extracellular matrix synthesis, angiogenesis	Activates tyrosine kinase receptors (FGFR) and stimulates MAPK and PI3K/Akt pathways	Agrawal et al. (2021), Hanneken et al. (2021), Liu et al. (2020c), Ma et al. (2019), Mossahebi-Mohammadi et al. (2020), Song and Finley (2020)
TGF-β (Transforming Growth Factor beta)	Promotes extracellular matrix synthesis, inhibits cell proliferation, induces apoptosis (at high concentrations)	Activates serine/threonine kinase receptors (TGF-βR) and stimulates SMAD pathways	Grewal et al. (2023), Jia et al. (2020), Longenecker et al. (2021), Riching et al. (2021), Waghabi et al. (2022)
HGF (Hepatocyte Growth Factor)	Promotes cell proliferation, cell migration, extracellular matrix synthesis	Activates tyrosine kinase receptors (c-Met) and stimulates MAPK and PI3K/Akt pathways	Guo et al. (2021), Harrington et al. (2023), Zhang et al. (2020)
EGF (Epidermal Growth Factor)	Promotes cell proliferation, cell migration	Activates tyrosine kinase receptors (EGFR) and stimulates MAPK and PI3K/Akt pathways	Qin et al. (2020), Ren et al. (2022), Sabbah et al. (2020), Sun et al. (2021b), Te Molder et al. (2021), Yuki, 2021; Zhang et al. (2024)

## 4 Application of amniotic membrane on cardiac disease

According to the World Health Organization (WHO), ischemic heart disease is a leading cause of death, claiming approximately 3.9 million lives annually. Recent studies suggest that amniotic membrane and stem cells may offer promising therapeutic options for treating ischemic heart damage and regulating inflammation (Table 4) (Tarin-Carrasco et al., 2021). The restoration of pathological ventricular function and improvement of ejection fraction are paramount goals in the treatment of heart injuries. The heart’s ventricle serves as the body’s primary blood pump, and damage to it can lead to heart failure, reduced quality of life, and even death. Improving ejection fraction refers to increasing the heart’s ability to pump blood, and both factors directly influence the heart’s function and its ability to supply blood to the body’s organs. If left untreated, heart injuries can result in chronic heart failure (Pilz et al., 2022). The treatment of ischemic heart damage and the regulation of inflammation by amniotic membrane and stem cells have been confirmed in recent years. Studies have shown that acellular human amniotic membrane (AHAM) and bone marrow mononuclear cells (BMMC) improve ejection fraction and enhance cardiac function within 30 days. Both BMMC and AHAM treatments led to improved ejection fraction and reduced pathological ventricular remodeling, indicating overall enhanced cardiac function. These improvements in cardiac function can be attributed to various factors, including the paracrine effects of stem cells, the anti-inflammatory properties of the amniotic membrane, and the combined effects of these factors (Takejima et al., 2024). A study investigated the protective effects of human amniotic membrane proteins (AMPs) on rat cardiomyocytes exposed to the anticancer drug doxorubicin (DOX). Researchers employed a multi-parametric assay to assess various parameters related to cellular injury, including intracellular Ca^2+^, ROS levels, antioxidant status, MDA, mitochondrial membrane potential, cell viability, and apoptosis. Results demonstrated that pretreatment with AMPs effectively mitigated DOX-induced toxicity in cardiomyocytes. AMPs significantly reduced elevated levels of LDH, Ca^2+^, ROS, and MDA while concurrently enhancing ΔΨm and antioxidant status. Furthermore, AMPs suppressed the expression of p53 and Bax proteins and attenuated NF-κB p65 activity, indicating their capacity to prevent oxidative stress and apoptosis. These findings suggest that AMPs hold promise as a therapeutic agent for preventing DOX-induced cardiotoxicity (Faridvand et al., 2020).

**TABLE 4:** Studies related to the application of amniotic membrane in cardiac tissue engineering.

Materials	Structure	Type	Effects	Ref.
hAM-BMMC	decellularization	*in vivo* (Rat)	↑ ejection fraction (EF) ↓ LVESV and LVED ↑ NF-kβ levels ↑ NALP3 enzyme levels	Takejima et al. (2024)
hAMSCs-PEG functionalized SPIONs	co-precipitation	*in vivo* (Rat)	↑ CD29, CD73, CD166 expression ↑ EF and fractional shortening (FS) ↓ TNF-α, IL-1β, TGF-β1, IL-6, IL-8, and CRP expression level ↓ fibrosis and abnormality ↓ phosphorylated p38 MAPK and NF-κB positive cells expression	Naseroleslami et al. (2021)
hAMSCs	decellularization	*in vivo* (Rat)	↑ EF and FS ↓ LVIDd and LVIDs ↓ fibrosis ↓ collagen deposition ↓ p53 and Bax protein expression ↑ Bcl-2 protein expression ↓ apoptotic index	Kheila et al. (2021)
hAMSCs	decellularization	*in vivo* (Rat)	↑ EF and FS ↑ systolic, diastolic, and mean arterial blood pressures ↑ LVSP ↓ LVEDP ↓ myocardial fibrosis ↓ collagen (I and III) deposition ↑ VEGF expression	Razavi Tousi et al. (2022)
hCardio and hAESC- amnion bilayer 3D scaffold	decellularization	*in vitro* (Rat)	↑ TRA-1-60, SSEA-4, Oct-3/4, Nanog, cTnT, adhesion molecule ICAM, PECAM+/VCAM- (endothelial marker) expression ↓ CD73, CD90, CD105 expression ↓ HLA-DR (0%), HLA-ABC (0.2%) immune antigens expression ↓ TNF-α levels (after 1-5-7-8 days of cultivation) ↑ TGF-β, GATA-4, Nkx-2.5, MEF-2C, myosin heavy chain (MHC), and α-actinin (ACTN2) expression	Normalina et al. (2022)
hAMSCs	decellularization	*in vitro*	↑ CD73, CD90, CD105 and CD44 expression ↓ CD19, CD11b, CD45, and HLA-DR expression ↑ MLC2v, Nkx2.5, and MyoD levels ↑ troponin T and α-actin expression	Shih et al. (2024)
hAMSCs-CM	decellularization	*in vivo* (Rat)	↑ SOD and GPx levels ↓ cTn-I and MDA expression ↓ myocardial injury ↑ TGF-β signaling ↓ IL-6, IL-1β, and TNF-α expression ↓ infarct size	Mokhtari et al. (2020)
hAMSCs-CM	decellularization	*in vivo* (Rat)	↑ EF and FS ↓ apoptotic nuclear density and fibrosis level ↑ angiogenesis ↑ CD29, CD105, and CD166 expression	Maleki et al. (2019)
hAM-ADMSCs	decellularization	*in vivo* (Rat)	↓ inflammation and fibrosis ↑ CD34^+^ expression ↑ angiogenesis ↓ CD45^+^ and CD68^+^ levels	Khorramirouz et al. (2019)
hAMP	decellularization	*in vitro*	↓ LDH, Ca^2+^, ROS, and MDA levels ↓ cell toxicity and apoptosis ↑ ΔΨm, SOD, CAT levels ↓ NF-kB p65 activity ↑ p53 and Bax protein levels	Faridvand et al. (2020)
PGS-PCL-hAMSCs	decellularization and copolymerization	*in vitro*/*in vivo* (Rat)	↑ cell survival ↑ EF, FS, stroke volume, LVEF, and EVD ↓ systolic volume ↑ VEGF expression	Bahrami et al. (2023)
hAM-PLGA	decellularization and electrospinning	*in vitro*	↑ endothelial cell viability, migration, and tube formation ↑ angiopoietin-1, VEGF-C, IL-8	Hasmad et al. (2022)
hAM-PCL-MoS_2_	decellularization and electrospinning	*in vitro*	↑ biocompatibility, elongated morphology and cell aggregation ↑ c-TnT, GATA-4, NKX 2.5, and α-myosin heavy chain expression	Nazari et al. (2022)
hAM	decellularization	*in vitro*/*in vivo* (Rat)	↑ biocompatibility ↑ LVEF and LVFS ↓ infarct size	Henry et al. (2020)
hAM-BMSCs	decellularization	*in vivo* (Rat)	↑ EF, LVEDV and LVESV ↑ desmine-positive cells ↑ angiogenesis ↑ connexin-43 expression	Blume et al. (2021)
hAM-(15d-PGJ_2_) nanoparticles	decellularization and nanoprecipitation	*in vivo* (Rat)	↑ cell viability ↑ LVEF ↓ infarct size ↑ connexin-43 and CD31 expression	Francisco et al. (2020)

**FIGURE 3:**
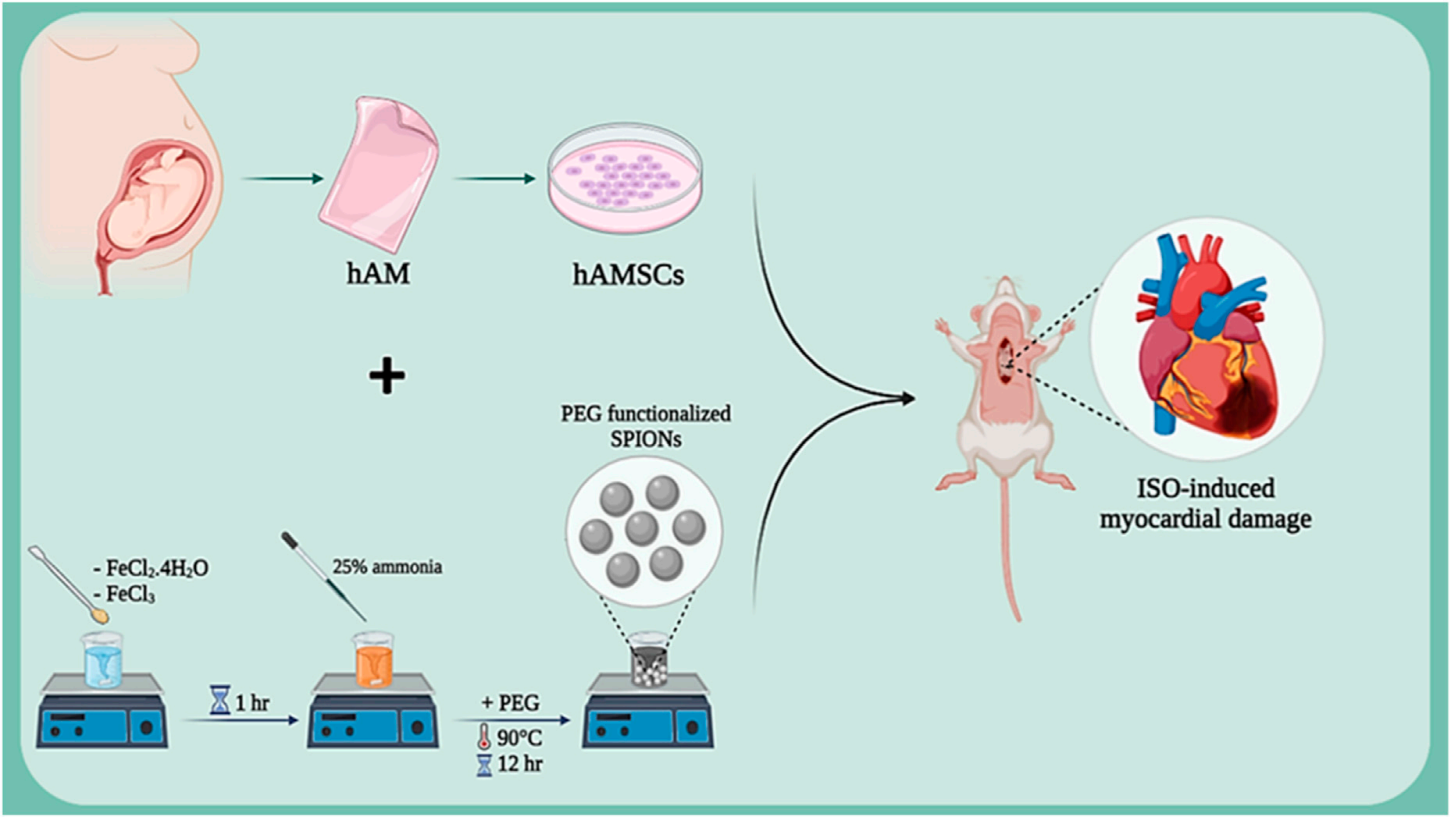
Schematic illustration of SPION-labeled hAMSCs preparation for ISO-induced myocardial damage. Created with BioRender.com.

Isoproterenol (ISO), as a beta-adrenergic agonist, induces myocardial damage by overstimulating cardiac beta-adrenergic receptors. This damage manifests as increased heart rate, blood pressure, and myocardial oxygen consumption, ultimately leading to ischemia and necrosis of cardiac cells. Studies have shown that isoproterenol is a suitable model for studying ischemic heart damage and evaluating various therapeutic effects (Akumwami et al., 2024; Fan et al., 2020). In various studies, this method has been used to assess the regenerative capacities of amniotic membrane in heart injury repair. In one study, the protective effects of isoproterenol (ISO)-induced myocardial damage were investigated using human amniotic membrane mesenchymal stem cells (hAMSCs) labeled with superparamagnetic iron oxide nanoparticles (SPION) (Figure 3). Results showed that SPION-labeled hAMSCs in the presence of a magnetic field can control inflammation through the NF-κB/MAPK pathway, consequently improving cardiac function and reducing fibrosis and tissue damage. This positive effect is due to the high capacity of hAMSCs to migrate to the damaged area of the heart and secrete anti-inflammatory factors (Naseroleslami et al., 2021). Another study examined the protective effects of human amniotic membrane-derived mesenchymal stem cells (hAMSCs) on ISO-induced myocardial injury. By secreting growth factors and anti-inflammatory cytokines, hAMSCs can prevent mitochondrial damage, inhibit cytochrome C release, and suppress the apoptotic process. Results showed that transplantation of hAMSCs after myocardial injury can increase cardiac dimensions and restore fractional shortening (FS) and ejection fraction (EF). Additionally, hAMSCs, by affecting the intrinsic (mitochondria-dependent) apoptotic mechanism and upregulating Bcl-2 expression, reduced ISO-induced myocardial injury. Furthermore, myocardial interstitial fibrosis was decreased with hAMSC transplantation (Kheila et al., 2021). In another study, the effect of hAMSCs transplantation on cardiac fibrosis in an ISO-induced heart failure model was investigated. Results showed that hAMSCs transplantation could reduce cardiac fibrosis, decrease the deposition of collagen types I and III, and increase VEGF expression. These improvements led to improved myocardial structure and cardiac function in the heart failure model. This positive effect is due to the ability of hAMSCs to reduce inflammation, enhance angiogenesis, and decrease collagen deposition. (Razavi Tousi et al., 2022).

**FIGURE 4:**
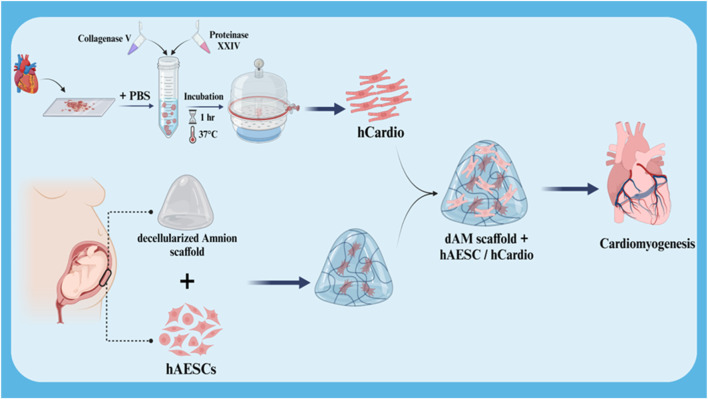
Schematic illustration of co-culture of hCardio + hAESC in decellularized Amnion 3D scaffold for cardiomyogenesis. Created with BioRender.com.

The differentiation of stem cells into cardiac cells is a novel approach in the treatment of heart injuries. This process involves culturing stem cells with high proliferation capacity and the ability to differentiate into various cell types in a suitable culture medium to differentiate them into cardiac cells. These differentiated cardiac cells are then transplanted into the damaged area of the heart. The newly formed cardiac cells can replace the damaged cells and improve heart function. The importance of this approach lies in the fact that stem cells can be used as an endless source for the production of new cardiac cells and can also be genetically manipulated to perform better. In addition, stem cells can secrete growth factors and cytokines that help repair heart tissue and reduce inflammation (Deszcz, 2023; Foo et al., 2021; Kawaguchi and Nakanishi, 2022). In a study, the effect of the co-culture ratio of human amniotic mesenchymal stem cells (hAESC) and human cardiac cells (hCardio) on cardiac cell differentiation in a 3D matrix used for cardiac patches was investigated (Figure 4). The results showed that a 6:1 ratio (hAESC to hCardio) is optimal for cardiac cell differentiation from hCardio. At this ratio, the differentiated cardiac cells showed expression of genes related to cardiac differentiation, such as cTnT and ACTN2. Also, the differentiated cardiac cells in this ratio showed migration and protopodia formation (Normalina et al., 2022).

Another study aimed to chemically induce human amniotic membrane mesenchymal stem cells (hAMSCs) into cardiomyocyte-like cells. Results showed that hAMSCs isolated from amniotic membrane samples expressed surface markers characteristic of mesenchymal stem cells and exhibited a trilineage differentiation potential into adipocytes, chondrocytes, and osteoblasts. The markers SSEA-1, SSEA-3, and SSEA-4 were also positive. Using various chemical induction methods, hAMSCs were successfully induced to express cardiac-specific genes such as MLC2v, Nkx2.5, MyoD, troponin T, and α-actin. These results suggest that hAMSCs can be chemically manipulated to differentiate into cardiomyocyte-like cells *in vitro*. However, further research is necessary to optimize induction methods and improve the functional properties of the differentiated cells (Shih et al., 2024).

In recent years, various studies have focused on the application of the amniotic membrane in different types of heart injuries to better understand its potential in cardiac injuries. Mokhtari et al. investigated the cardioprotective effects of human amniotic membrane mesenchymal stem cells (hAMSCs)-conditioned medium (CM) on rat cardiac ischemia/reperfusion (I/R) injury. A myocardial infarction (MI) model was created and treated with either culture medium or hAMSCs-CM. They aimed to determine whether substances secreted by hAMSCs could help protect the heart from damage caused by reduced blood flow and oxygen supply. Results showed that hAMSCs-CM significantly increased the activities of antioxidant enzymes (SOD and GPx), reduced oxidative stress markers (cTn-I and MDA), and improved cardiac histological alterations. These findings suggest that hAMSCs-CM may be a promising therapeutic option for I/R injury by reducing oxidative stress (Mokhtari et al., 2020). In another study, the effects of mesenchymal stem cells derived from human amniotic membrane (MSC-CM) on heart failure (HF) in rats were investigated. Rats were induced to develop HF and then treated with MSC-CM, culture medium, or PBS. Results showed that MSC-CM significantly improved heart function, reduced fibrosis, and increased angiogenesis, suggesting its potential as a therapeutic agent for HF (Maleki et al., 2019). The efficacy of amniotic membrane (AM) in treating myocardial infarction lesions was also investigated. After inducing a myocardial infarction model, rats were treated with a patch containing adipose-derived mesenchymal stem cells (ADMSCs) seeded on a decellularized human AM. Results showed that the patch-implanted group had less inflammation, fibrosis, and apoptosis (Khorramirouz et al., 2019).

**FIGURE 5:**
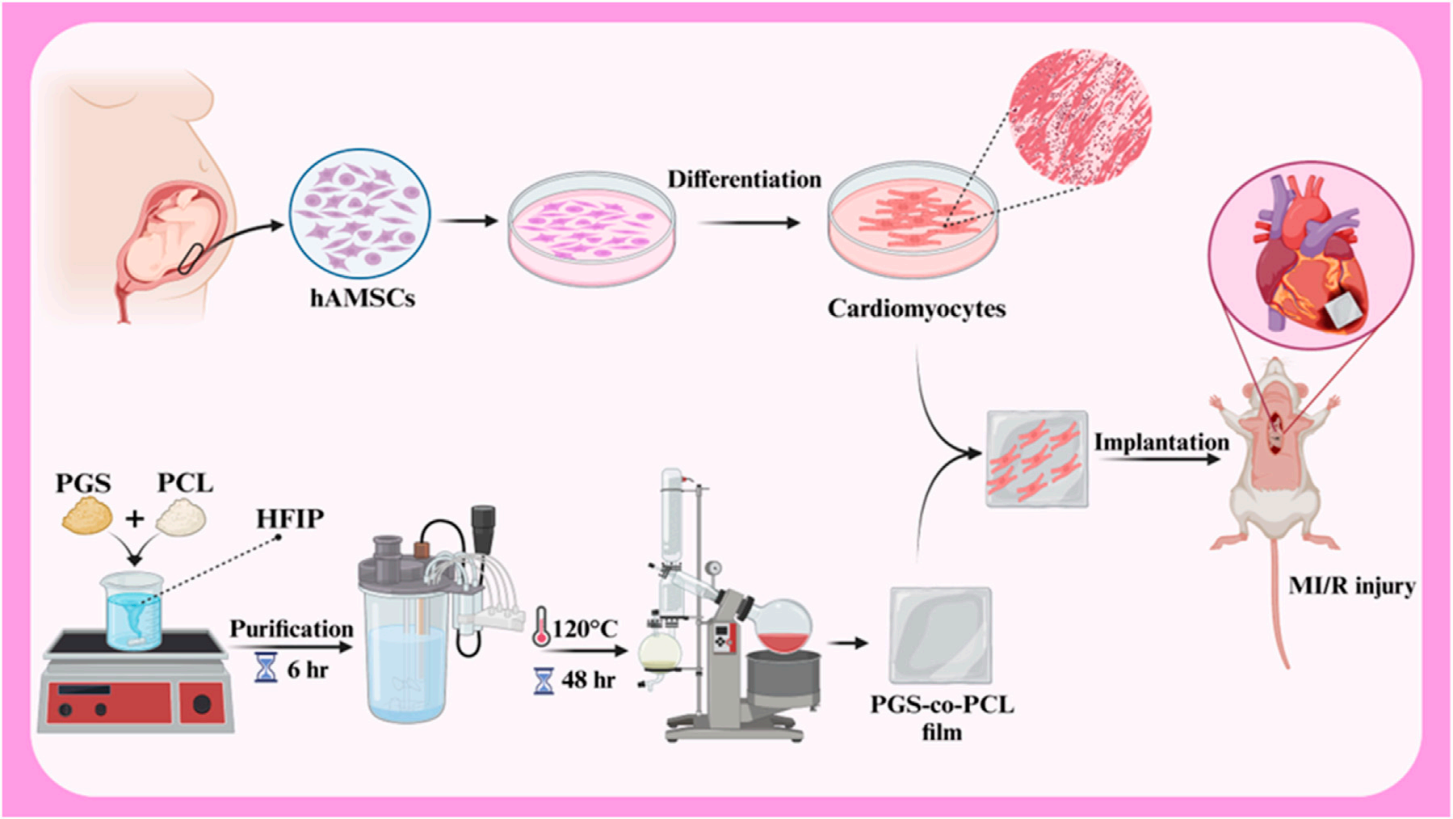
Schematic illustration of hAMSCs differentiation to cardiomyocytes, PGS-co-PCL cardiac films preparation, and implantation of PGS-co-PCL film + differentiated hAMSCs to MI/R injury rat model. Created with BioRender.com.

hAMSCs play a positive role in the repair of myocardial ischemia-reperfusion injury (MI/R) due to the stimulation of endogenous repair mechanisms. A study investigated the effects of a combined therapy using human amniotic membrane mesenchymal stem cells (hAMSCs) and a tissue-engineered film based on poly glycerol sebacate (PGS)-co-polycaprolactone (PCL) on myocardial ischemia-reperfusion injury (MI/R) in rats (Figure 5). *In vitro* results showed good cell survival on the films, and *in vivo* results demonstrated improved cardiac function, including increased fractional shortening, stroke volume, LVEF, and end-diastolic volume. The study also showed increased expression of VEGF protein, suggesting that the combined therapy may enhance angiogenesis. These findings suggest that the combined treatment of hAMSCs and cardiac film is a promising therapeutic approach for MI/R injury (Bahrami et al., 2023). In another study investigated the potential of cardiac patches based on human amniotic membrane (hAM) coated with electrospun polylactic-co-glycolic acid (PLGA) fibers for cardiac regeneration. Conditioned medium (CM) obtained from these patches was cultured on human umbilical vein endothelial cells (HUVECs) to assess their pro-angiogenic properties. The results showed that CMs derived from hAM-PLGA scaffolds had increased levels of pro-angiogenic factors, including VEGF-C, IL-8, and angiopoietin-1, and promoted better endothelial cell viability, migration, and tube formation compared to CMs derived from plain hAM. These findings suggest that cardiac patches based on hAM-PLGA could be a promising therapeutic option for ischemic injuries by promoting angiogenesis (Hasmad et al., 2022).

**FIGURE 6:**
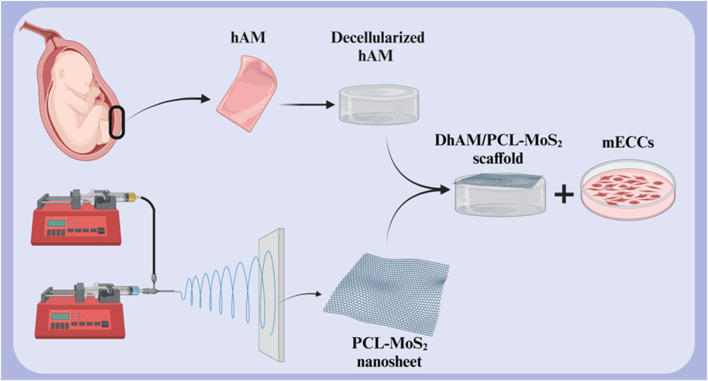
Schematic illustration of **(A)** decellularized hAM scaffold, **(B)** electrospun PCL-MoS_2_ nanosheet, and **(C)** DHAM/PCL-MoS_2_ scaffold for cardiac tissue engineering. Created with BioRender.com.

One of the primary challenges in cardiac tissue engineering is restoring the natural electrical conductivity of the heart following injury. Cardiac cells require electrical communication with each other to function synchronously. Cardiac tissue engineering scaffolds, in addition to providing an environment for cell growth and proliferation, must also facilitate electrical conductivity. Scaffolds with high electrical conductivity, such as those containing conductive nanoparticles, can improve communication between cardiac cells, thereby restoring cardiac electrical function and improving contractility. This is particularly important in the treatment of diseases such as myocardial infarction, which is associated with impaired electrical conduction (Chen et al., 2020; Edrisi et al., 2023; Ul Haq et al., 2021). A study investigated the potential of a novel scaffold based on decellularized human amniotic membrane (DHAM) coated with molybdenum disulfide (MoS2) and PCL nanosheets for cardiac tissue engineering (Figure 6). The results showed that the scaffold was biocompatible and supported the growth and maturation of mouse embryonic cardiac cells (mECCs). The presence of cardiac genes such as c-TnT, GATA-4, NKX 2.5, and α-myosin heavy chain in mECCs cultured on the DHAM/PCL-MoS2 scaffold suggests that it can enhance the differentiation of mECCs into cardiomyocytes. These findings indicate that DHAM/PCL-MoS2 scaffolds may be a promising candidate for cardiac tissue engineering applications (Nazari et al., 2022). In 2019, Henry et al. introduced a novel hAM-based injectable matrix to promote post-MI cardiac regeneration. After injection of hAM matrix into the heart of the MI mouse model by ultrasound guidance, fibrosis was significantly reduced, and cardiac contractility and EF were improved. The results of this study showed that the design of an injectable hAM matrix and its potential effectiveness can play a major role in cardiac regeneration (Henry et al., 2020).

## 5 Clinical translation and prospects

**FIGURE 7:**
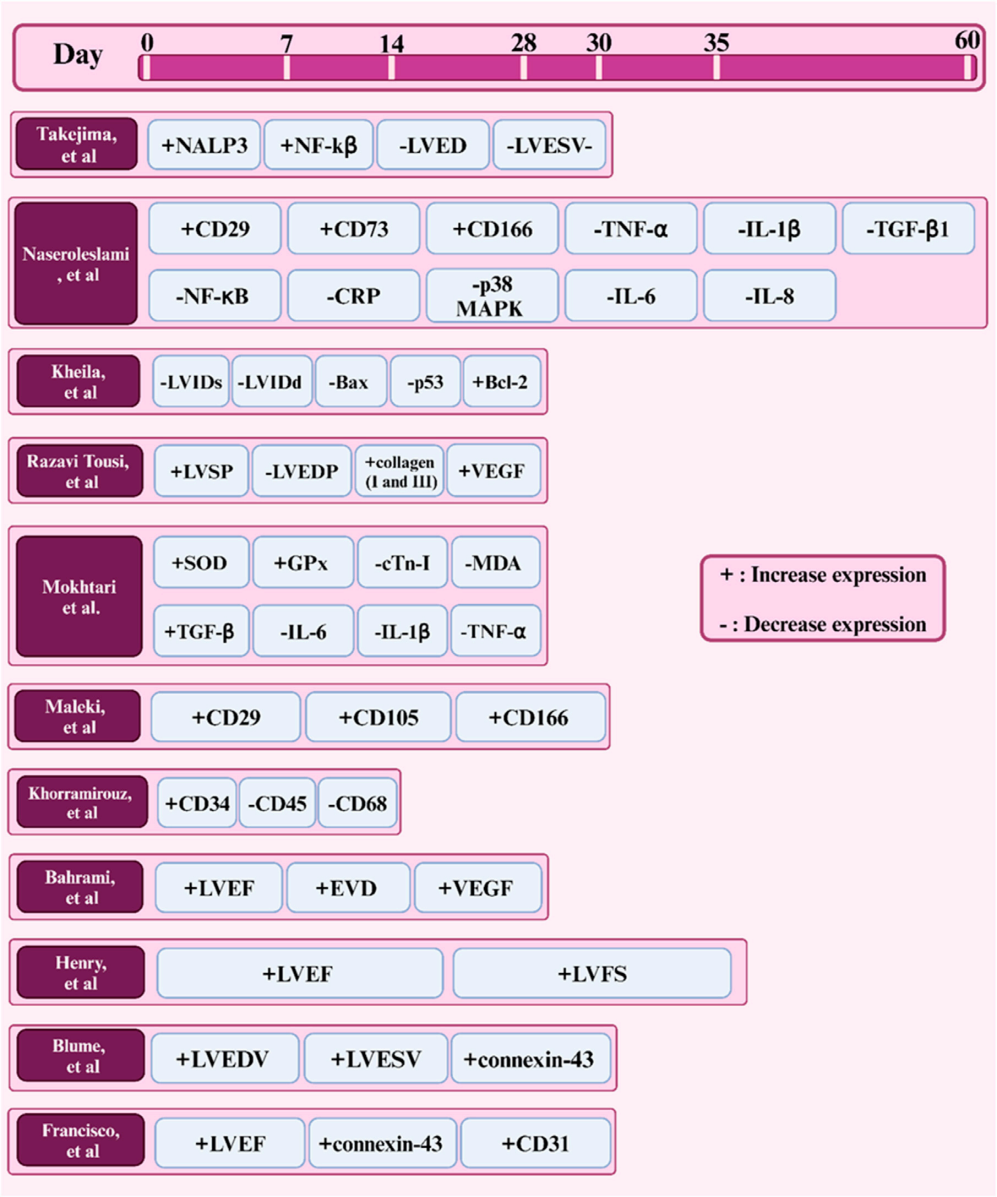
Timeline of factors expressed in studies (*in vivo*) of the amniotic membrane in cardiac tissue engineering.

Amniotic membrane (AM) has emerged as a potential and promising biomaterial for cardiac tissue engineering due to its regenerative capabilities and unique properties such as anti-inflammatory activity and biocompatibility. Although significant progress has been made in this area, several key topics require further research. One of the new, widely used, and cost-effective techniques for obtaining AM scaffolds is decellularization. However, modifications can be made to this technique to improve and develop more efficient and reproducible methods for preserving desired ECM components while minimizing immunogenic complications. In addition, decellularization compounds and various agents can be investigated to better remove ECM from other cell components. To match the mechanical properties of AM with native cardiac tissue, techniques such as incorporation of conductive materials, cross-linking, or combining AM with other biomaterials can be used to create hybrid scaffolds with improved elasticity and mechanical strength properties. Furthermore, exploring techniques to enhance the differentiation of cardiac progenitor cells or stem cells into cardiomyocytes after culture on AM scaffolds may improve cell-AM interactions. In this way, the combination of biological molecules such as growth factors can promote cell adhesion, proliferation, and differentiation in AM scaffolds. To date, the results of implanting AM-based constructs in small animal models have been promising Figure 7, while preclinical studies have been conducted in large animal models to evaluate the safety and long-term effectiveness of AM-based cardiac patches, as well as to develop effective designs, conduct, and controlled clinical trials to evaluate AM -based treatments in patients with heart failure may be a way forward. Combination therapies would be another solution to promote heart repair and regeneration. In this context, the combination of AM scaffolds with immunomodulatory treatments or drug delivery systems can be mentioned to improve graft survival and function. On the other hand, the use of patients’ cells and biomaterials as well as the use of computer models to predict the behavior of AM-based cardiac patches in the complex cardiac environment can revolutionize the personalization of treatment approaches in cardiac patients. By exploring these future prospects, researchers can realize the potential of the amniotic membrane as a versatile platform for cardiac tissue engineering and develop more innovative treatments for cardiac patients.

## 6 Conclusion

Cardiovascular diseases are among the most significant global health challenges. Despite significant advancements in the treatment of these diseases, there is still a need for novel approaches to repair damaged cardiac tissue. Amniotic membrane, as a biological scaffold with unique properties, has shown great potential in cardiac tissue repair and improving cardiac function. Its unique properties such as biocompatibility, anti-inflammation, and wound healing stimulation have made this membrane a promising option for the treatment of heart diseases. Numerous studies have shown that amniotic membrane, containing growth factors, cytokines, and other biomolecules, can stimulate tissue repair processes, reduce inflammation, and enhance angiogenesis. These properties have made amniotic membrane a promising option for the treatment of various heart diseases, including myocardial infarction, heart failure, and congenital heart diseases.

Although the results of initial studies have been promising, larger-scale clinical studies are still needed to fully determine the long-term efficacy and safety of using amniotic membrane in the treatment of heart diseases. Additionally, standardization of amniotic membrane preparation and processing methods, individual variations in amniotic membrane composition, the need for more clinical studies to determine the optimal dose and application method, and the potential risk of disease transmission are among these challenges.

Overall, amniotic membrane has great potential to improve treatment outcomes in heart patients. However, for the widespread use of this method in the clinic, further studies and standardization of the production and application process are needed. Additionally, interdisciplinary collaboration between cardiologists, tissue engineers, and biologists is essential for developing new applications of amniotic membrane. In the future, with advancements in manufacturing technologies and tissue engineering, amniotic membrane can be expected to become an effective and safe therapeutic tool for cardiac tissue repair.
